# IoT-Based Precision Irrigation System Featuring Multi-Sensor Monitoring and Scheduled Automated Water-Control Gates for Rice Production

**DOI:** 10.3390/s26092692

**Published:** 2026-04-26

**Authors:** Mir Nurul Hasan Mahmud, Younsuk Dong, Md Mahbubul Alam, Jinat Sharmin

**Affiliations:** 1 Irrigation and Water Management Division, Bangladesh Rice Research Institute, Gazipur 1701, Bangladesh; nurul.iwm@brri.gov.bd (M.N.H.M.); mahbubul.iwm@brri.gov.bd (M.M.A.); jinat.iwm@brri.gov.bd (J.S.); 2 Biosystems and Agricultural Engineering, Michigan State University, East Lansing, MI 48824, USA

**Keywords:** IoT automated irrigation, alternate wetting and drying (AWD), soil moisture-based control, water use efficiency, rice cultivation

## Abstract

**Highlights:**

**What are the main findings?**

- An IoT-based precision irrigation system integrating soil moisture and water-level sensors with scheduled sensor-based automation effectively controlled AWD irrigation without manual intervention.
- The IRRI35 treatment maintained high grain yield (7.76 t ha^−1^) while reducing water use by 28%, energy consumption by 37%, and significantly improving water use efficiency compared to continuous flooding.

**What are the implications of the main findings?**

- Automated irrigation can overcome key adoption barriers of AWD by minimizing labor requirements and improving water governance.
- Large-scale implementation could reduce irrigation energy demand, and enhance sustainability in rice production systems.

**Abstract:**

Despite its significant water-saving potential, the adoption of alternate wetting and drying (AWD) irrigation remains limited due to infrastructure constraints and intensive manual monitoring requirements. An automated precision irrigation system was developed and tested at the Bangladesh Rice Research Institute research farm in Gazipur, Bangladesh. The system combined ultrasonic water-level sensors, capacitive soil moisture sensors, an Arduino-based microcontroller, a GSM communication module, and solar-powered automatic control gates. Field performance was evaluated following a Randomized Complete Block Design (RCBD) under four irrigation treatments: IRRISAT, IRRI35, IRRI25, and continuous flooding (CF). The first three irrigation treatments were operated using scheduled daily decision windows, in which irrigation actions were automatically triggered based on predefined schedules and sensor threshold values. In IRRISAT, irrigation started when soil moisture dropped slightly below saturation and stopped at a ponding depth of 5 cm, while IRRI35 and IRRI25 were triggered at volumetric soil water contents of 35% and 25%, respectively, with the same upper cutoff of 5 cm ponding depth; CF served as the control. The IRRI35 treatment achieved a high grain yield (7.76 t ha^−1^) while reducing water use by 28% and energy consumption by 37% compared to CF. Water use efficiency was considerably higher under IRRI35 (9.4 kg ha^−1^ mm^−1^) than under CF (6.7 kg ha^−1^ mm^−1^). The automated system proved to be reliable and precise in scheduled irrigation control, significantly reducing water use and labor requirements. The findings suggest that large-scale adoption of the system under real-world cultivation conditions could reduce irrigation energy needs and contribute to sustainable water governance in rice production.

## 1. Introduction

Bangladesh is an agrarian country, with agriculture the primary source of income for most of the population. Rice is the primary crop that is grown on nearly 80% of the total cropped area [1]. However, the country faces a severe water crisis during dry season due to groundwater depletion, declining surface water availability, and inefficient irrigation systems [2,3,4,5]. Inefficient water management leads to water waste, lower crop yields, and food insecurity [6]. Moreover, the erratic rainfall patterns due to climate change further exacerbate the water challenge [7].

Inefficient irrigation systems reduce productivity, as excessive water use can cause waterlogging, creating favorable conditions for insect and pest infestation [8]. In addition, traditional irrigation practices rely heavily on manual labor, resulting in significant time demands and higher costs. However, the agricultural sector is currently facing a labor shortage [9], driven by an aging workforce and declining interest in farming among younger generations. Therefore, there is a need to develop technologies that can automate water application in rice fields.

The increasing scarcity of surface water and declining groundwater resources can be mitigated through water-saving irrigation techniques such as Alternate Wetting and Drying (AWD). AWD allows the soil to become intermittently unsaturated during the rice-growing season, reducing irrigation water use by up to 38% without yield loss when properly implemented [10]. This method requires monitoring field water levels using a perforated tube installed in the soil, with irrigation applied when the water level falls 15–20 cm below the surface [11,12,13]. Consequently, irrigation timing depends on farmers’ manual observation of field water conditions.

Despite the water-saving potential of AWD, its adoption among farmers remains limited [14]. Several constraints hinder its effective implementation in farmers’ fields. Across South and Southeast Asia, adoption is constrained by a range of institutional and economic barriers, including limited infrastructure and extension support [15], fixed seasonal irrigation fees that discourage water saving [16], and a lack of direct incentives for saving water [17]. Sociocultural factors further hinder adoption, as farmers are often reluctant to frequently enter muddy fields to monitor water levels using tubes installed within plots [18]. In gravity-fed irrigation systems, water is supplied on a rotational basis, limiting farmers’ control over timing and often delaying irrigation beyond the optimal threshold [19].

Numerous studies have investigated automated AWD irrigation systems that utilize various sensors to measure ponding and perched water depth, enabling automatic start and stop of irrigation pumps based on predefined water thresholds. However, the large-scale deployment of sensor- and IoT-based technologies in integrated rice multi-field systems remains limited. Most existing research has focused on technical aspects, such as system architecture and component performance, often evaluated under laboratory conditions or in single-plot experimental setups. Alce et al. [20] developed an automated Safe AWD irrigation system for rice that employed ultrasonic and capacitive sensors to monitor field water level and soil moisture content. Rana et al. [21] proposed an automated alternate wetting and drying (AAWD) system in which sensor probes installed inside an AWD pipe regulated irrigation based on predefined upper (3 cm above the soil surface) and lower (10 cm below the soil surface) water thresholds. Similarly, Tolentino et al. [22] designed and evaluated an IoT-based automated irrigation and paddy water level control system following the AWD principle. In their system, an ultrasonic sensor (HC-SR04) housed inside a cylindrical PVC tube measured paddy water levels while minimizing interference from surface debris, and irrigation was controlled through automated gate operation using a stepper motor. While these studies demonstrate the technical feasibility of sensor-based AWD automation, they are predominantly limited to individual plot-level control and do not fully address the challenges of centralized pump operation, coordinated water distribution, and synchronized irrigation management across multiple plots under actual field conditions.

Thus, the goal of this study is to develop an IoT-based automated AWD irrigation system designed for multi-plot field conditions, where individual plots are irrigated through automated gates and integrate centralized pump control. This system enables coordinated irrigation management across multiple plots while maintaining plot-specific water regimes, addressing key limitations of existing plot-level automation approaches. The hypothesis of this study is that multi-plot IoT-based AWD irrigation system, which integrates real-time soil moisture and water level monitoring with scheduled sensor-based automated irrigation control, can improve water productivity, reduce irrigation water input, and decrease energy use compared with conventional flooding (CF) under rice cultivation. Furthermore, it is hypothesized that optimizing soil moisture trigger thresholds for irrigation can further enhance yield and resource-use efficiency. The specific objectives of this study were to:

(i) develop and implement a field-scale, multi-plot automated AWD irrigation system in which individual plots are managed via automated gates and all plots are supplied by a centralized pump,
(ii) evaluate the effects of different soil moisture trigger thresholds on rice yield, irrigation water use, and energy consumption compared with conventional flooding, and
(iii) assess the potential of the system as a practically scalable precision irrigation solution for improving water and energy productivity in rice-based systems.

## 2. Materials and Methods

### 2.1. Laboratory Experiment

**Figure 1:**
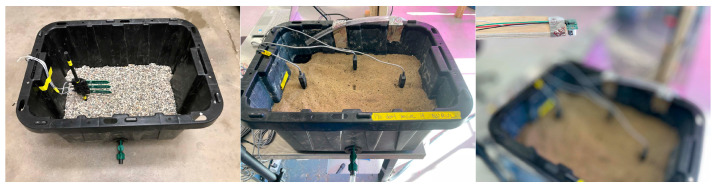
Physical model for water level and soil moisture monitoring.

Automated irrigation system construction, code development, and sensor calibration procedures were performed at the laboratory before deployment in the field. A plastic sandbox (45 cm × 45 cm × 60 cm) filled with pea gravel and field soil collected from the South Campus Farm, East Lansing, MI, USA were used for laboratory experimental setup (Figure 1). The ultrasonic sensor was installed above the sandbox to measure water depth and soil moisture sensors (SoilWatch 10) were installed within the soil profile.

### 2.2. Calibration of a Low-Cost Capacitive Soil Moisture Sensor

**Figure 2:**
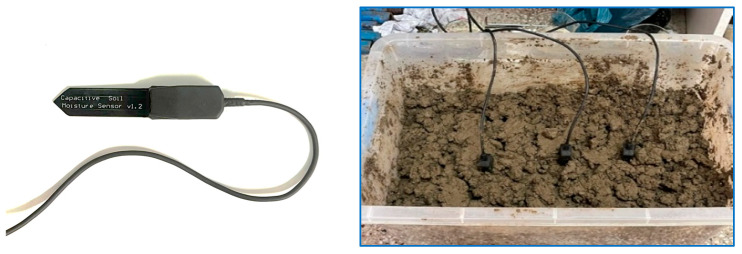
Calibration of low-cost capacitive soil moisture sensor (V1.2).

SoilWatch 10 sensor (Pino-tech, Zachodniopomorskie, Poland), a capacitance-based soil moisture sensor, was utilized in this study. The capacitance-type soil moisture sensor measures the dielectric constant of soil and water. Since its electrodes do not directly contact the soil, it is more resistant to corrosion than resistance-type sensors [23]. The sensor consists of a positive plate, a negative plate, and an intervening dielectric layer. The significant difference in dielectric constants between soil and water allows the sensor to detect water molecules in the soil and provide real-time data for precision irrigation management [24]. This sensor is cost-effective, energy-efficient, and reasonably accurate, making it common soil moisture sensor in smart and automated irrigation systems. The supply voltage requirement for SoilWatch 10 ranges from 3.3 to 5 V, which allows for integration with low-voltage microcontrollers [25]. Given the frequent soil saturation and ponding in rice cultivation, the sensor’s exposed circuitry was encapsulated with a rubber coating to prevent water damage and ensure reliable performance in flooded conditions (Figure 2).

Sensor calibration was conducted at the Bangladesh Rice Research Institute (BRRI) Irrigation Laboratory to establish the relationship between soil moisture sensor readings and the actual volumetric soil water content (VWC). The soil was collected from the BRRI research farm, and the soil type was loam. A known volume of air-dried soil (15,000 cm^3^) was placed in a 51 × 31 × 23 cm container (Figure 2). Water was then added incrementally in sixteen steps, with volumes ranging from 2200 to 6700 cm^3^ at 300 cm^3^ intervals. After each addition, the soil was thoroughly pulverized to ensure uniform moisture distribution, and the sensor readings were recorded. The VWC was calculated as the volume of water divided by the volume of soil and expressed as a percentage. The calibration curve was then included in the programming codes.

The low-cost soil moisture sensors were installed in the field and evaluated for performance under real-world conditions. 128 sensor data were recorded from 15 days after transplanting to the panicle initiation stage. Soil samples were collected using a 98.2 cm^3^ core ring and analyzed by the oven-dry method to determine the true VWC. The comparison between sensor readings and oven-dry data was used to assess sensor accuracy using three statistical metrics: Index of Agreement (IA), Root Mean Squared Error (RMSE), and Mean Bias Error (MBE). A detailed description of the sensor calibration procedure is provided by previous studies [24,26].

### 2.3. Field Experiment and Irrigation Treatments

The performance of an automated precision irrigation system was evaluated through a field experiment. Four treatments, including IRRISAT, IRRI35, IRRI25, and Continuous Flooding (CF), were tested in this study. Under IRRISAT, irrigation was automatically triggered when soil water content fell slightly below saturation and stopped when the ponded water depth reached 5 cm above the soil surface. For IRRI35 and IRRI25, irrigation was triggered when VWC declined to 35% and 25%, respectively, with the same upper cutoff of 5 cm ponding depth. Continuous flooding served as the control. During the early growing season, a ponding depth of 5 cm was maintained from the day of transplanting to 15 days after transplanting, and again from the panicle initiation stage to the ripening stage. By contrast, under conventional CF, irrigation was applied at regular intervals, maintaining a ponding water depth of about 5 cm from transplanting until the milk stage, which is commonly practiced by farmers.

### 2.4. Experimental Design and Plot Layout

**Figure 3:**
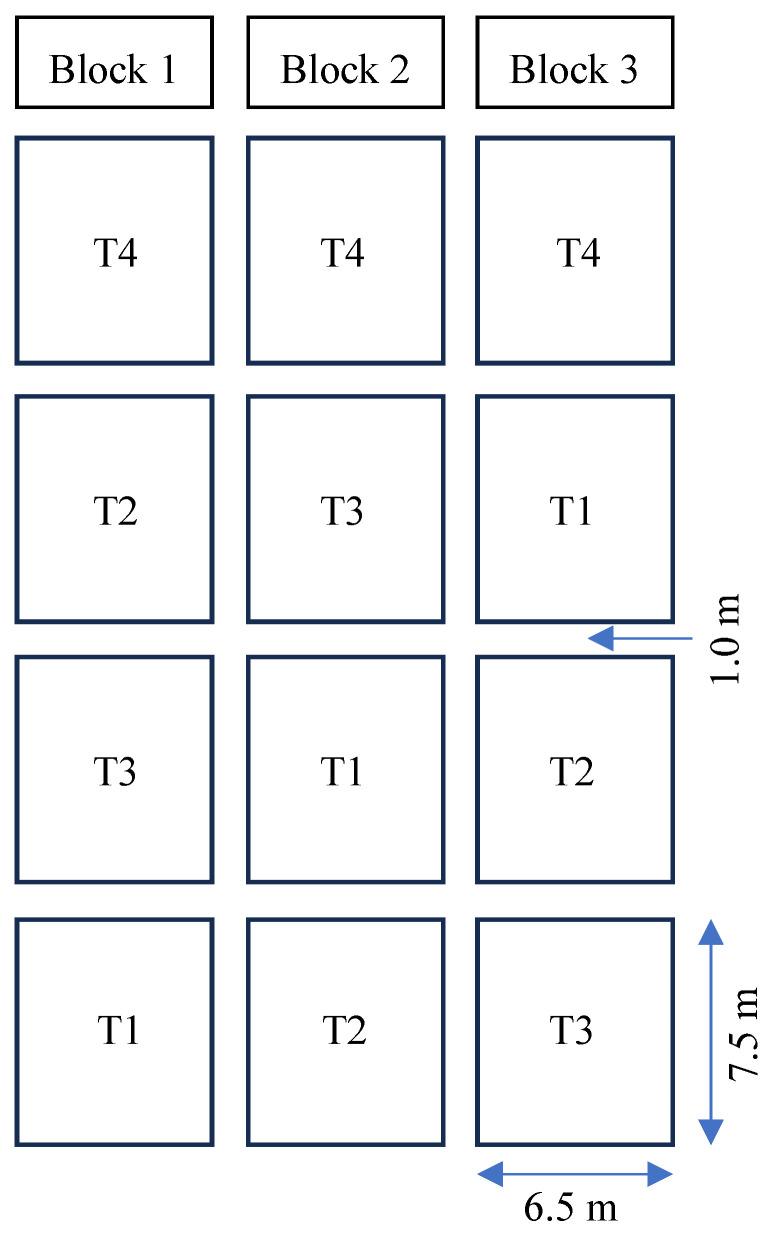
Field experiment layout at the BRRI research field.

The experiment was conducted with three distinct blocks, each serving as a replication (Figure 3). A buffer zone of about 1.0 m separated adjacent blocks to facilitate movement and minimize lateral water flow. Each block was divided into four treatment plots, each measuring 7.5 m by 6.5 m. The automated precision irrigation treatments were arranged randomly within each block. However, for operational convenience and management efficiency, the CF treatment was placed in the same row across blocks. The experimental field was specifically dedicated to irrigation water management research and was carefully prepared to ensure uniformity. Prior to the experiment, the field was leveled and managed to minimize variation in soil texture, fertility, and hydraulic properties. As a result, field conditions were relatively homogeneous, which likely reduced spatial variability across plots.

### 2.5. Site Description and Crop Management

**Figure 4:**
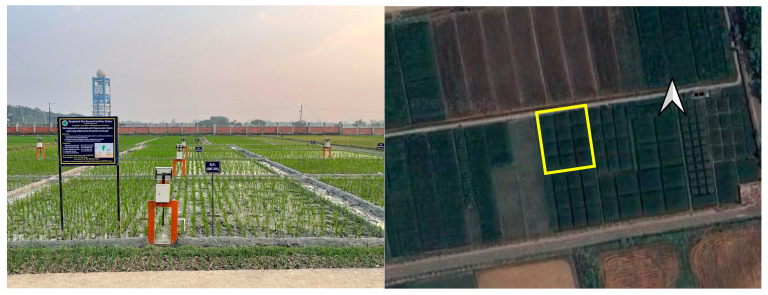
Field trial of the automated precision irrigation system (**left**). The yellow box represents the experimental field containing twelve plots (**right**).

The experiment took place during the Boro (cool dry) season of 2025 at the BRRI research farm in Gazipur, Bangladesh (23°59′22″ N, 90°24′13″ E; 7.0 m above mean sea level) (Figure 4). The soil at the experimental site was classified as loam. The region experiences a subtropical monsoon climate, characterized by moderately high temperatures and heavy rainfall during the summer (monsoon) season, and relatively lower temperatures with limited rainfall during the winter (dry) season. The volume of irrigation water applied to each plot was measured using a flowmeter. The irrigation water was delivered to the experimental field through a 7.6 cm hose pipe with no conveyance loss. Monthly rainfall was recorded throughout the experimental period using observations from the BRRI weather station The rice variety BRRI dhan89, with a long growth duration of 155 days, was used in this study. Forty-day-old seedlings were transplanted on 18 January 2025. All plots received identical management, including recommended doses of fertilizers, weeding, herbicide application, and pest control, in accordance with BRRI guidelines. Harvesting was carried out on 12 May 2025.

### 2.6. Data Collection and Statistical Analysis

Plant growth characteristics and yield were measured in a designated sampling area of approximately 1.0 m^2^ of each treatment, and grain yield was determined from a 10 m^2^ sampling area within each plot and adjusted to 14% moisture content. The experiment was laid out in a Randomized Complete Block Design (RCBD) with treatments as fixed effects and blocks as random effects. The statistical model used was $Y_{ij}=\mu +\tau_{i}+\beta_{j}+\epsilon_{ij}$, where $Y_{ij}$ is the observed response, μ the overall mean, $\tau_{i}$ the treatment effect, $\beta_{j}$ the block effect, and $\epsilon_{ij}$ the random error. Normality of residuals was tested using the Shapiro–Wilk test, and homogeneity of variance was checked using Levene’s test; both assumptions were satisfied. Mean differences between treatments were compared using the Least Significant Difference (LSD) test at the 5% significance level. All analyses were performed using R and RStudio (Version 4.3.1).

### 2.7. System Hardware and Irrigation Logic Development

**Figure 5:**
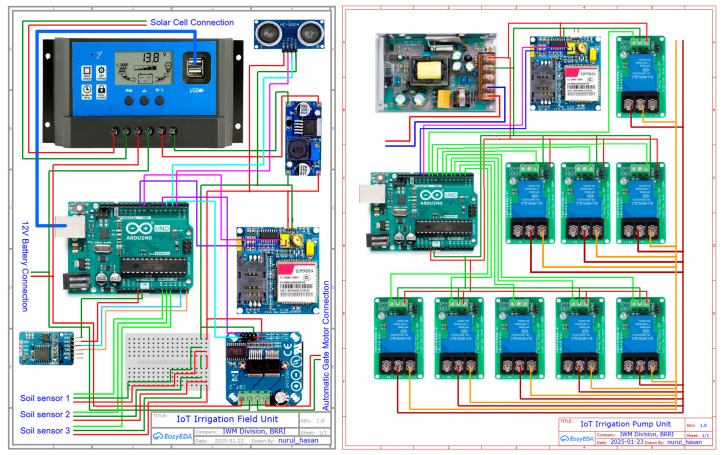
Circuit diagram of the field unit (**left**) and the pump unit (**right**). In the field unit, the red wires are for the 5V power supply, dark green wires are for the ground connections, and the light green wires are input connections from the sensors to the Arduino board. In the pump unit, the dark brown and the orange wires connected to the relays are for live (220 V) and ground connections, respectively.

An IoT-based precision irrigation system was developed using an ultrasonic water-level sensor, three capacitive soil-moisture sensors at depths of 0–5, 5–10, and 10–15 cm, an Arduino Uno microcontroller, a GSM/GPRS SIM module (SIM900A), a real-time clock module (DS3231), a 12 V solar panel, and a rechargeable battery. These components are to develop an IoT and sensor control panel (Field unit), the automatic water gate, and the pump control unit (Figure 5). A 220 V, 1.5 kW submersible pump with a maximum water discharge of 230 L/min was used in this experiment.

**Figure 6:**
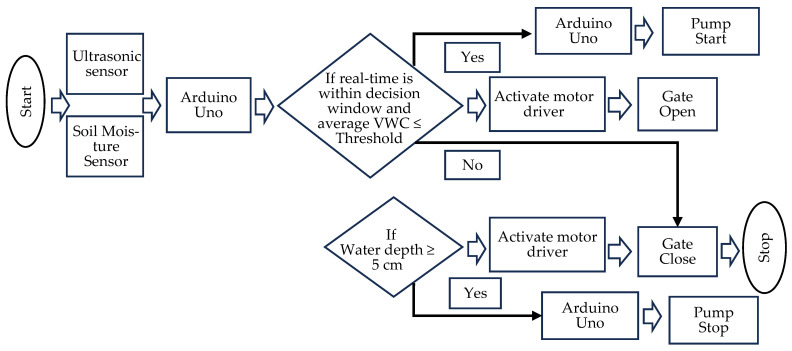
Flow chart of the automatic basin irrigation system.

The Arduino Uno board transmitted data via the SIM900A module over a Grameenphone 2G mobile internet connection to a cloud server (Thingspeak) for storage and visualization. The Arduino Uno board processed real-time sensor data locally using programmed code that followed predefined daily schedules (decision windows) to make irrigation decisions. Irrigation decisions were also based on the average VWC of three sensors installed at depths of 0–5, 5–10, and 10–15 cm. Based on these decisions, the system generated output commands, such as activating the DC motor mounted on the automatic gate, and sent an SMS command to the pump unit (Figure 6). Another Arduino Uno board in the pump unit received SMS commands from the field unit to turn on the automatic pump. An integrated approach was implemented to coordinate irrigation across multiple rice plots, ensuring efficient and synchronized water management. Before deployment, the capacitive soil moisture sensor was calibrated in the laboratory for sixteen moisture levels.

#### 2.7.1. Irrigation Control Gate Automation and DC Motor Function

The irrigation control gate was automated using a sensor-based control logic integrated with a DC motor-driven linear actuator. Gate operation was determined by predefined VWC and water depth thresholds programmed into the control unit. Irrigation was applied when VWC decreased to 25%, 35%, or near saturation. This triggered the gate to open and terminated when the ponded water depth reached 5 cm, at which point the gate closed.

**Figure 7:**
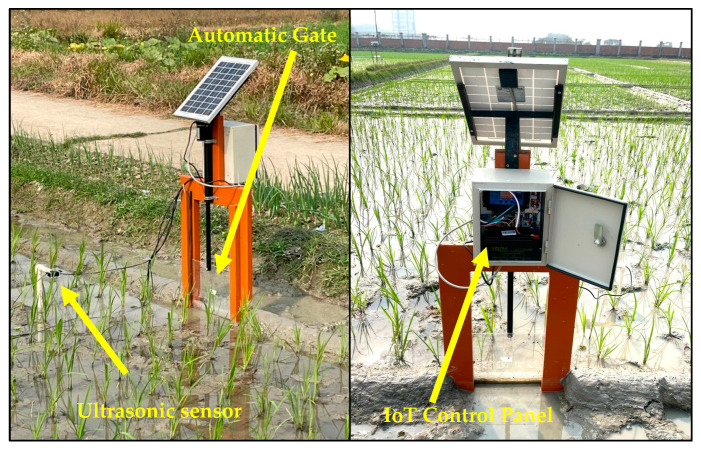
Field Setup of the experiment at the BRRI research field showing the sensor, IoT control panel, and automatic water gate deployment. The transparent shutter of the automatic gate is in the closed position.

A DC motor-driven telescopic linear actuator was used to control gate operation (Figure 7). The gate was interfaced with a motor driver module (HW-039) that enabled bidirectional rotation, allowing controlled extension and retraction of the actuator shaft for opening and closing the gate. Each opening and closing operation takes approximately 11 s. The gate shutter was fabricated from a plastic sheet mounted on a wooden frame and guided vertically by a U-channel mechanism to ensure stable, aligned movement. The gate assembly was installed at the field inlet to regulate irrigation inflow.

#### 2.7.2. Irrigation Scheduling Using Real-Time Clock (RTC) Module

Irrigation scheduling was implemented using a DS3231 RTC integrated with the microcontroller. The control algorithm operated on a time-triggered polling system, making irrigation decisions at two fixed times each day (10:00 a.m. and 4:00 p.m.), rather than using continuous real-time control. During each scheduled period (10:00–11:00 a.m. and 4:00–5:00 p.m.), the system evaluated real-time VWC data collected from sensors installed in the field. Irrigation was initiated when the average VWC dropped below preset threshold levels. The RTC module ensured precise timing for decision-making and prevented unscheduled pump operation. In addition to irrigation scheduling, the RTC module was used to calculate and log days after transplanting (DAT) for system monitoring and data management.

#### 2.7.3. Pump Unit Configuration and Control Mechanism

The pump unit was installed adjacent to the water source and the main electrical supply and was designed to operate the irrigation pump based on remote commands received from the field control unit. The system consisted of an Arduino Uno microcontroller, a SIM900 GSM module for communication, a 5 V DC power supply, 30 W relay switches, and a 1.5 kW submersible pump (Figure 5).

Irrigation commands generated by the field unit were transmitted via SMS using the SIM900 GSM module. Upon receipt of a command, the Arduino Uno processed the signal and actuated the relay switch to start the pump. Pump operation continued until a stop command was received, which was triggered by sensor-based feedback on ponded water depth at the field unit. The relay switches were selected to match the electrical load requirements of the 1.5 kW pump. Water was delivered from the source to the experimental plots through earthen conveyance canals. A single-phase digital electric energy meter was installed on the pump circuit to measure the electrical energy consumption (kWh) by the pump motor for each irrigation event done to each experimental plot and converted to kWh ha^−1^.

#### 2.7.4. Centralized Pump Control for Multiple Field Units

Each irrigation treatment plot was equipped with an independent autonomous field unit. An integrated control logic was developed to coordinate irrigation requests from multiple field units with a single centralized pump unit. Despite the use of a centralized pump unit, each experimental plot maintained independent irrigation control through plot-specific sensors and control gates, with sequential execution of irrigation events ensuring treatment independence. Each field unit evaluated VWC and water depth conditions independently and transmitted irrigation commands to the pump unit via SMS.

When two or more plots required to apply irrigation simultaneously, the corresponding controls gates were opened, and each field unit sent an “ON” command to the pump unit. For example, if plots 2, 4, and 5 required irrigation, their respective gates were opened, and field units 2, 4, and 5 sent “ON” commands, activating Relay 2, Relay 4, and Relay 5 in the pump unit; while plot 2 was being irrigated, the control gates of plots 4 and 5 remained open to allow immediate continuation of irrigation without interrupting pump operation. As one plot finished its irrigation, the corresponding field unit sent an “OFF” command to deactivate only its specific relay. For instance, once plot 2 completed irrigation, Relay 2 was deactivated while Relays 4 and 5 remained active. In this setup, the pump kept running as long as at least one relay remained active. Once the final plot (plot 5) completed irrigation and sent its “OFF” command, the last active relay (Relay 5) was turned off, which caused the system to stop the pump. This logic ensured that irrigation was plot-specific and responsive, preventing premature shutdowns while allowing shared use of a single pump.

A single Arduino microcontroller controlled the nine relay modules (one channel each) at the pump unit (Figure 5). The power supply was properly sized to power all relays and the SIM900 GSM module without any shortages. Throughout the Boro 2025 season, it was observed that no more than three plots needed irrigation on the same day, reducing system load and ensuring efficient operation. Additionally, one plot (7.5 m by 6.5 m) took about 15 min to irrigate, and the next plot was irrigated without delay in the pump operation.

### 2.8. Cloud Server Integration

The ThingSpeak cloud server served as the data storage and visualization platform for remote monitoring of field conditions. Each field unit was configured to transmit up to four parameters, comprising one ponded water-level measurement and three soil-moisture measurements. Data transmission was carried out via GPRS using the SIM900 GSM module integrated with the Arduino-based field units.

Sensor data were uploaded to the ThingSpeak server at predefined intervals, typically within 30 s of acquisition. The cloud platform stored and displayed time-series data for VWC and ponding depth, enabling remote access to field condition information without requiring on-site presence. ThingSpeak was selected for its compatibility with SIM900-based Arduino systems, which enabled direct data transmission without additional gateways or external data processing platforms.

### 2.9. Manual Perched Water Level Observations

Manual measurements of water levels were taken intermittently throughout the growing season to capture the perched water table below the water surface at each VWC threshold. In each plot, a perforated PVC observation pipe (10 cm diameter, 55 cm length) was installed to a depth of 45 cm. Water levels within the pipes were recorded immediately before irrigation using a steel ruler, providing reference data for different irrigation regimes.

### 2.10. Data Transmission Performance Evaluation

The performance of the communication system was evaluated based on both GPRS-based data transmission and GSM/SMS-based control events. The number of successfully received GPRS data packets was obtained from the datasets downloaded from the ThingSpeak cloud platform. These records were used to quantify the actual number of data transmissions successfully uploaded during the experimental period.

The total number of expected (generated) data transmissions was estimated based on the duration of the experiment, data transmission interval, and the number of deployed sensors. The system operated continuously for 100 days, with data transmission at 30-s intervals across nine plots. The expected number of transmissions was calculated accordingly and compared with the received dataset to determine the transmission success rate and failure percentage.

For GSM-based communication, the total number of SMS commands was calculated based on the number of irrigation events recorded during the experiment. Each irrigation event generated two SMS commands: one for pump activation (start) and another one for pump deactivation (stop). The total number of issued SMS commands was then compared with the number of successfully received and executed commands to evaluate SMS communication reliability.

This combined approach enabled a comprehensive assessment of communication performance, including data transmission efficiency and overall system responsiveness under field conditions.

## 3. Results and Discussion

### 3.1. Field Performance of the Soil Moisture Sensor

**Figure 8:**
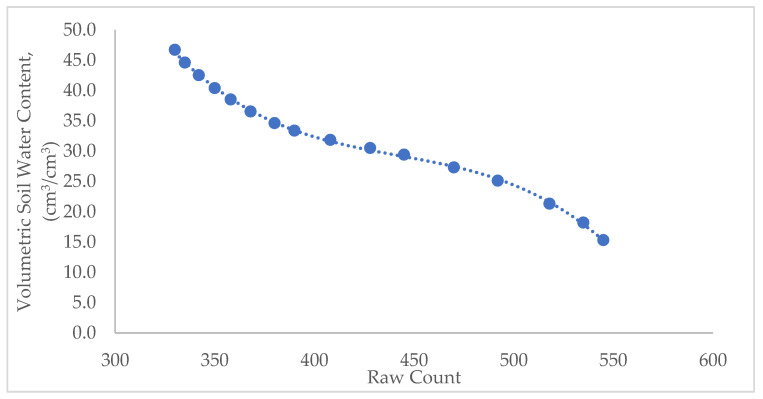
Calibration of the low-cost capacitive soil moisture sensor (V1.2) in the laboratory.

The calibration curve, derived from the results, established a relationship between the sensor output (analog raw count) and the actual VWC, enabling accurate estimation of soil moisture under field conditions (Figure 8). In contrast to other soil sensors, the VWC and voltage exhibited an inverse relationship, indicating that higher voltage corresponded to lower moisture content [24]. This calibration curve was integrated into the control codes uploaded to the Arduino microcontroller at the field unit. The calibration of the low-cost capacitive soil moisture sensor encompassed a broad VWC from 15 to 47%, enabling the embedded calibration equation (Equation (1)) to automatically convert real-time sensor readings into actual VWC percentages for irrigation control and data logging during the field experiment.

(1) $$VWC\%=-7\times 10^{-6}{RC}^{3}+9.6\times 10^{-3}\;{RC}^{2}-4.30\;RC+682.31$$

where VWC% represents the volumetric soil water content as a percentage, and RC is the analog raw counts, which are the outputs from the sensor.

**Figure 9:**
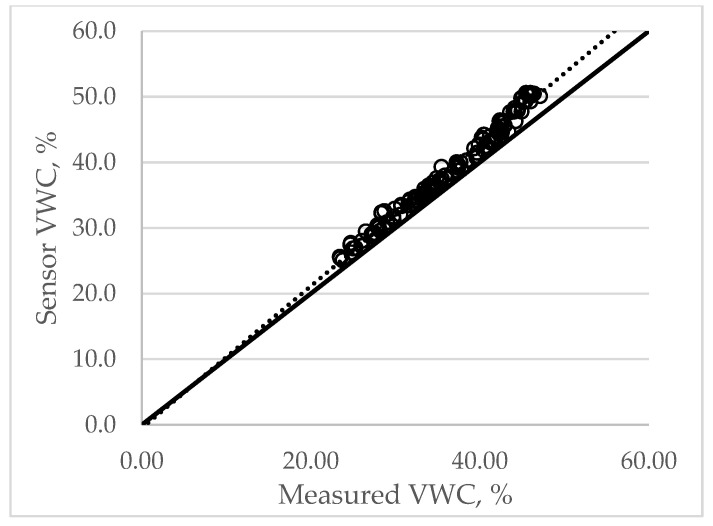
Field performance evaluation of the soil moisture sensor. The solid line is a 1:1 line, and the dotted line drawn as the trend of the circles is the line to compare between the measured and sensor VWCs.

The Index of Agreement (IA) was 0.96, indicating excellent correspondence between the sensor readings and the gravimetric measurements (Figure 9). The Root Mean Squared Error (RMSE) was 0.027, reflecting minimal random error and confirming that the sensors maintained consistent precision across varying VWC levels. The Mean Bias Error (MBE) was 0.01, indicating a slight positive bias. These results suggests that the sensors marginally overestimated VWC by approximately 1–1.5% at VWC below 40% and by 2–3% at VWC above 40%, compared with the oven-dry method. Such a small bias is common for low-cost capacitive soil moisture sensors and can be corrected through calibration if higher measurement accuracy is required.

During the study period, sensors were operated under standard irrigation and flooding conditions, and no operational failures were observed. While the sensors performed reliably throughout the experiment, long-term durability, drift under prolonged flooding, and performance across other soil types were not evaluated in this study.

### 3.2. Communication Reliability and Operational Performance of the Automated Irrigation and Gate Control System

The communication performance of the developed irrigation system was evaluated over a continuous 100-day field experiment, during which sensor data were transmitted at regular intervals via the Grameenphone 2G GSM/GPRS network to the ThingSpeak cloud platform. A total of approximately 10.29 million GPRS transmission events were generated during the whole season (Table 1). Of these, 9.86 million transmissions were successfully received, corresponding to an overall success rate of 95.84%, while approximately 0.43 million transmissions (4.16%) experienced temporary failures.

Transmission failures did not happen continuously but appeared as short interruptions, usually lasting 1–2 min each. These occurred about 20–30 times per day mostly around midday. This timing likely relates to peak network congestion, higher user traffic, and signal changes typical to 2G communication systems. On average total downtime was about 55 min per day, matching the calculated failure rate and showing that the communication losses were brief, not ongoing.

Even with these interruptions, the system kept working because it used a GSM reset mechanism every 30 s. This feature allowed the system to recover quickly from communication stalls, preventing long disconnections and reducing data loss. As a result, the system remained highly reliable during real-world field tests, despite temporary failures.

The communication latency was measured as well. The average GPRS transmission delay was between 17 and 30 s, and the average SMS response time was about 11 s. These results match what is expected for GSM/GPRS networks, where latency depends on network load, signal strength, and data processing. The level of delay is acceptable for irrigation management, since real-time response is not essential.

The control system’s reliability was also tested using SMS command. During the experiment, 210 SMS commands were sent, all were received and carried out successfully, No commands were missed. This shows that the control layer is highly reliable, which is important for use in the field.

Overall, with the Grameenphone 2G network, the SIM900 module maintained over 95% uptime. This allowed steady SMS-based control and regular data updates to the ThingSpeak platform with few communication problems. The system was accurate, responsive, and stable, making it suitable for fully automated irrigation.

The automatic gate mechanism responded accurately to sensor commands according to the set irrigation logic (Figure 6). It achieved a 100% operational success rate because it was directly connected to the controller with wires. There was no physical obstruction, mechanical jamming, or operational failure during gate’s opening and closing cycles, and no water leaked when the gate was closed. The system worked reliably throughout the experiment, which added to the effectiveness and strength of the automated irrigation system.

### 3.3. Selection and Justification of Soil Moisture Thresholds for AWD Irrigation

The principle of safe AWD irrigation is that when the perched water depth declines to 10–15 cm below the soil surface, the soil matric potential reaches approximately −10 to −20 kPa [27,28]. For loam soils, this matric potential range typically corresponds to a VWC of about 38–40% at −10 kPa and 33–36% at −20 kPa, based on established soil water retention relationships reported in the literature [29,30]. In addition, the IRRISAT treatment was designed to initiate irrigation when soil water content fell slightly below saturation, corresponding to approximately 42.5% VWC, which is close to the saturated water content of loamy soils. In this study, the 35% VWC threshold was selected to represent a condition within the safe AWD range, while a lower threshold of 25% VWC was used to represent a comparatively drier soil condition beyond the conventional AWD range, allowing evaluation of more water-saving irrigation strategies. These thresholds were derived from published soil hydraulic characteristics and AWD guidelines, rather than site-specific soil water retention measurements.

While this approach provides a reasonable approximation for loam soils, the absence of site-specific soil water retention measurement introduces uncertainty in the relationship between VWC and soil matric potential under field conditions. Soil variability may affect the exact threshold levels at which safe AWD occurs. Therefore, the selected thresholds in the present study should be considered as generalized reference values, and future work should focus on site-specific soil water retention measurement to improve precision and broader applicability.

### 3.4. Rice Yield Contributing Characters

Irrigation treatments significantly affected tiller and panicle densities, whereas grains per panicle and thousand-grain weight (TGW) were unaffected (Table 2). The highest tiller (281.0 m^−2^) and panicle (261.0 m^−2^) densities were recorded under IRRI35, which was statistically superior to CF and IRRI25. IRRISAT produced an intermediate response, particularly for panicle density (254.3 m^−2^), which did not differ significantly from IRRI35 but was higher than CF and IRRI25. The lowest numbers of tillers (249.7 m^−2^) and panicles (232.0 m^−2^) were observed under IRRI25, indicating a reduced capacity for tiller production and survival under this irrigation regime.

These findings indicate that irrigation management primarily affected early vegetative growth and tiller survival, both of which are highly sensitive to soil moisture. The superior performance of IRRI35 is consistent with previous research, demonstrating that AWD can enhance tiller productivity in rice relative to CF, likely due to improved soil aeration and a more favorable root environment under intermittent moist conditions [31]. In contrast, the reduced tiller and panicle densities observed under IRRI25 likely results from increased moisture stress, leading to higher tiller mortality and fewer effective panicles. The intermediate performance of IRRISAT indicates its potential to sustain productive tiller density with optimized water use, which aligns with reports that well-managed non-continuous irrigation can enhance root activity and tiller productivity in rice [32].

In contrast, grains per panicle and TGW were not significantly affected by irrigation treatments, with grains per panicle ranging from 130.7 to 133.0 and TGW from 23.6 to 23.8 g. This indicates that reproductive development and grain filling were relatively stable across treatments once panicles were established. The uniformity in grains per panicle and TGW suggests that water availability during the flowering and grain-filling stages was not limiting, as a continuous water depth of 5 cm was maintained after the heading stage. This is consistent with previous findings showing that the irrigation method often has little impact on these reproductive traits when adequate water is supplied during the panicle initiation and grain-filling stages [33,34].

### 3.5. Rice Yield, Total Water Use, and Water Use Efficiency (WUE)

**Figure 10:**
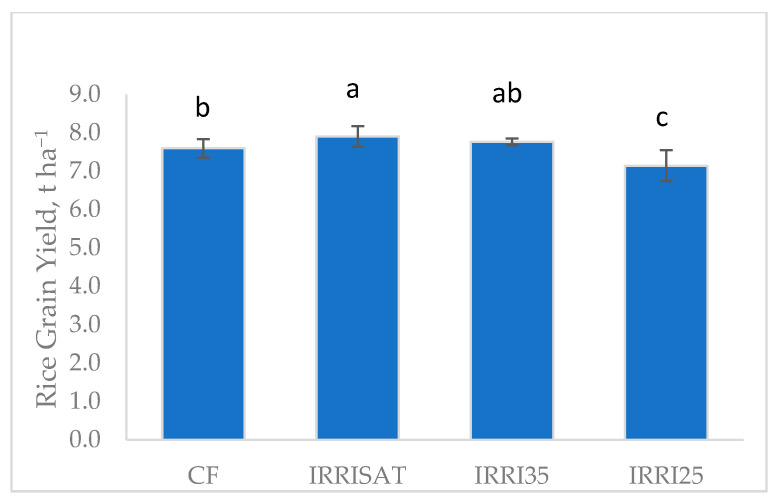
Mean rice grain yield of BRRI dhan89 achieved at different irrigation treatments. Common letters between yields of two treatments do not differ statistically at either the 1% or 5% level of probability (analyzed using R).

Analysis of variance showed a highly significant effect of irrigation treatments on grain yield, total water received, and WUE (*p* < 0.01). Grain yield ranged from 7.14 to 7.90 t ha^−1^, reflecting clear treatment effects. The highest yield was achieved under IRRISAT (7.90 t ha^−1^), which was significantly greater than that of CF (7.59 t ha^−1^) (Figure 10). The fixed (non-randomized) placement of the CF treatment is a limitation of the experimental design. Although field conditions were relatively homogeneous, this does not fully substitute for randomization; potential positional effects cannot be entirely excluded when interpreting treatment comparisons. Yield under IRRI35 (7.76 t ha^−1^) was statistically comparable to IRRISAT, whereas IRRI25 (7.14 t ha^−1^) produced the lowest yield, indicating that more severe soil drying can adversely affect yield.

The yield response closely mirrored trends in tiller and panicle density, which were significantly higher under IRRISAT and IRRI35 than under CF and IRRI25 (Table 3). These results indicate that the superior yields achieved under IRRISAT and IRRI35 were primarily driven by the production and survival of a greater number of productive tillers and panicles, rather than changes in grains per panicle or thousand-grain weight, which remained statistically unchanged across treatments. These results are consistent with reports that soil moisture regimes significantly influence tiller and panicle formation, which in turn contribute to final grain yield [35].

Irrigation treatments also strongly influenced total water input and WUE. Continuous flooding required the highest total water input (1142 mm), whereas IRRI25 had the lowest (732 mm). Correspondingly, WUE was lowest under CF (6.7 kg ha^−1^ mm^−1^) and highest under IRRI25 (9.7 kg ha^−1^ mm^−1^), followed by IRRI35 (9.4 kg ha^−1^ mm^−1^). Compared with CF, IRRISAT and IRRI35 reduced water input by approximately 20% and 28%, respectively, demonstrating substantial water-saving potential.

However, although IRRI25 maximized WUE, its lower grain yield indicates that excessive soil drying limited tiller survival and panicle formation, as shown by the reduced tiller and panicle densities under this treatment. In contrast, IRRI35 achieved a more favorable balance between water savings and yield, maintaining high tiller and panicle densities while significantly reducing irrigation input. While IRRISAT produced marginally higher yields, IRRI35 is recommended as the preferred treatment because it offers a multi-objective tradeoff by maintaining nearly maximum yield while conserving water. This strategy supports with the principle of optimizing both productivity and resource conservation, rather than selecting treatments based solely on maximum yield.

These findings corroborate previous studies indicating that AWD can reduce irrigation water use by approximately 20 to 40% and improve WUE without substantial yield loss [34]. However, severe soil drying during AWD may decrease yield, highlighting the importance of establishing appropriate soil moisture thresholds to balance water savings with crop performance [33,35,36].

### 3.6. Energy Savings for Irrigation

Irrigation management had significant impact on energy consumption (*p* ≤ 0.01). Continuous flooding required the highest energy input (932.5 kWh ha^−1^), highlighting its intensive resource use (Table 4). In contrast, precision irrigation treatments remarkably reduced energy use. The IRRISAT treatment lowered energy consumption to 695.8 kWh ha^−1^, representing a 25.4% reduction compared to CF. Greater reductions were achieved with IRRI35 (588.0 kWh ha^−1^; 36.9% reduction) and IRRI25 (497.5 kWh ha^−1^; 46.6% reduction), with IRRI25 exhibiting the lowest energy requirement. These results suggest that precision irrigation strategies conserves water and significantly decrease the energy required for field operations, thereby improving overall system efficiency. Previous field studies have shown that AWD and similar precision irrigation methods can reduce irrigation hours and fuel/electricity needs for pumping, further emphasizing the energy and cost advantages over traditional flooding [37].

From farmers’ perspectives in shared pump systems, the observed energy savings have economic significance but must be evaluated alongside yield responses. Under continuous flooding, the high energy requirement (932.5 kWh ha^−1^) corresponds to an electricity cost of approximately 4900 BDT ha^−1^ (40 USD ha^−1^) at BDT 5.25 per kWh. Adoption of IRRISAT reduced energy use to 695.8 kWh ha^−1^, lowering irrigation costs to about 3650 BDT ha^−1^ (30 USD ha^−1^) and saving 1250 BDT ha^−1^ (10 USD ha^−1^) without notable yield reduction. Greater savings were achieved under IRRI35 and IRRI25, with irrigation costs declining to approximately 3100 BDT ha^−1^ (26 USD ha^−1^) and 2600 BDT ha^−1^ (22 USD ha^−1^), representing savings of 1800 BDT ha^−1^ (14 USD ha^−1^) and 2300 BDT ha^−1^ (18 USD ha^−1^), respectively. However, while IRRI25 maximized energy savings (46.6%), it also produced the lowest grain yield, with a yield penalty of 0.45 t ha^−1^ compared to CF, equivalent to 11,000 BDT ha^−1^ (90 USD ha^−1^). This reduces its overall economic appeal under shared pump conditions where yield stability is essential, particularly for risk-averse smallholders. In contrast, IRRI35 achieved a more favorable balance by delivering substantial energy savings (36.9%) while maintaining relatively higher yields. This approach reduces collective pump runtime, alleviates peak-time irrigation conflicts, and enhances cost sharing among users. Overall, the findings suggest that while severe soil moisture thresholds enhance energy efficiency, moderate precision irrigation (IRRI35) is likely the most practical option, as it aligns energy cost reduction with yield preservation and operational feasibility in communal pumping systems.

Reductions in energy use may have significant implications for greenhouse gas (GHG) emissions. Lower energy consumption can decrease indirect CO_2_ emissions associated with irrigation pumping, as carbon emissions from irrigation are primarily driven by energy use during water extraction and conveyance [38]. Water-saving irrigation methods such as AWD, which are conceptually similar to the precision irrigation strategies examined in this study, have also been shown to mitigate methane (CH_4_) emissions from rice fields. For instance, Islam et al. [39] reported that AWD irrigation reduced cumulative CH_4_ emissions by approximately 37% across multiple sites in Bangladesh. Although GHG emissions were not directly measured in the present study, existing evidence suggests that precision irrigation may improve the environmental sustainability of rice production by potentially reducing both direct (CH_4_) and indirect (CO_2_) emissions [40].

### 3.7. Soil Drying Dynamics and Irrigation Scheduling Under Intermittent Irrigation

**Figure 11:**
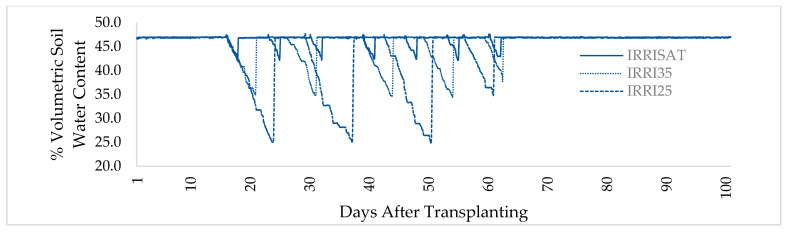
Volumetric soil water content as measured by capacitive soil moisture sensors under three irrigation treatments (IRRISAT, IRRI35, and IRRI25), averaged across three sensors placed at 0–5, 5–10, and 10–15 cm soil depths.

**Figure 12:**
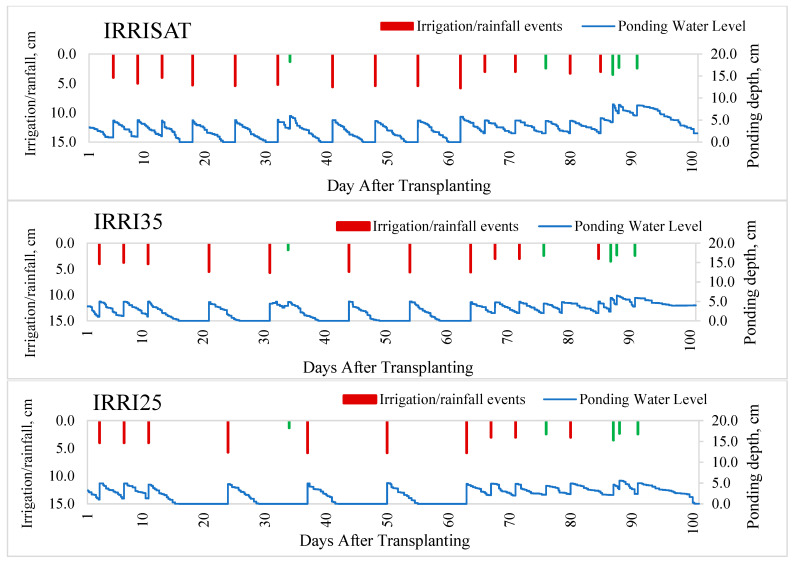
Ponding water depth as measured by the ultrasonic sensor under three irrigation treatments (IRRISAT, IRRI35, and IRRI25). Red vertical bars indicate irrigation, and green bars represent rainfall events.

The duration of soil drying periods under precision irrigation varied substantially among treatments, reflecting the specific VWC thresholds established by the sensor-based control strategy (Figure 11 and Figure 12, and Table 5). Drying periods, defined as the interval between the disappearance of ponded water from the soil surface and subsequent reflooding, ranged from 2 days under IRRISAT to 8 days under IRRI25. In contrast, CF maintained a ponding water depth of approximately 5 cm throughout the season, resulting in the highest irrigation frequency, with 16 irrigation events.

Intermittent irrigation regimes permitted progressive drying of the soil profile before re-irrigation, substantially reducing irrigation frequency. Under IRRISAT, irrigation was triggered when VWC in the 0–15 cm layer remained near saturation (approximately 42.5%), while the perched water table declined to 3–5 cm below the soil surface. This shallow drying produced 14 irrigation events, only slightly fewer than CF, but eliminated prolonged surface ponding. In IRRI35, allowing soil moisture to decrease to 35% VWC enabled the perched water table to recede to 19–22 cm, extending drying duration and reducing irrigation events to 11. The most pronounced drying occurred under IRRI25, where soil moisture depletion to 25% VWC permitted the water table to fall to 38–41 cm, extending drying periods to 8 days and reducing seasonal irrigation requirements to only 10 events. Decreasing soil moisture thresholds also affected re-saturation dynamics. The time required to re-saturate the 0–15 cm soil layer increased from 1.7 h under IRRISAT to 6.7 h under IRRI25. These results demonstrate that sensor-based precision irrigation effectively monitored soil drying and re-saturation cycles, while reducing irrigation frequency and enhancing water savings in irrigated rice. The system also provides a clear pathway to reduce water application while maintaining controlled soil moisture conditions, with potential applicability to non-rice crop cultivation.

### 3.8. Cost of the System

The cost of an automated irrigation system comprising an IoT and sensor control panel for each plot, an Automatic Irrigation Control Gate for each plot, and an Automatic Pump Control Unit is presented in Table 6. The total approximate cost of the field unit per plot was US$142, and the cost of the automatic pump control unit was US$30. When considering maintenance and communication expenses, the total cost of the automated irrigation system was US$184.

### 3.9. Breakeven Analysis

A preliminary estimate of the economic performance was conducted based on energy and labor savings. The annual energy cost saving under IRRI35 was approximately 14 US$ ha^−1^. Manual irrigation typically requires one laborer per irrigation event, and with around 16 irrigation events per season, the total labor cost for CF irrigation was estimated at 112 US$ ha^−1^ (US$7 per laborer per event). Automation effectively eliminates this labor requirement, increasing the total annual savings to approximately 126 US$ ha^−1^ when combined with energy savings.

The total cost of the field unit per plot was approximately US$142, while the automatic pump control unit cost US$30. Assuming that the pump control unit is shared among 10 plots, its cost is reduced to US$3 per plot. Therefore, including maintenance and communication expenses, the effective cost of the automated irrigation system was estimated at approximately 157 US$ per plot.

Based on these simplified assumptions, the preliminary payback period of the automated irrigation system is approximately 1.25 years (157 US$ ÷ 126 US$). The analysis is indicative and does not account for factors such as component replacement, battery and solar panel degradation, actuator wear, installation and training costs, technician support, or maintenance uncertainty over multiple seasons. Further evaluation under real operational conditions is needed to provide a robust assessment of economic feasibility.

## 4. Conclusions

The IoT-based real-time multi-sensor monitoring and scheduled sensor-based automated irrigation control demonstrated reliable and precise operation under real-world rice cultivation conditions. The system improved water use efficiency without reducing yield, particularly at the 35% VWC threshold and has the potential to reduce both water and energy use. Preliminary estimates indicate payback period of one to two years, suggesting promising economic feasibility, although further evaluation over multiple seasons is necessary. The system may also reduce reliance on manual labor, and future designs could primarily utilize soil moisture-based control, potentially omitting the ultrasonic sensor once reliable thresholds are established. Although the system was tested for only one season at one site, with one variety and one soil type, the results suggest potential applicability to other crops and environments. The non-randomized placement of the CF treatment is a design limitation of this study, and positional effects on yield may still influence the results. Extended multi-location trials and farmers’ training on this technology are needed to confirm large-scale performance, adoption potential, and broader impacts. Furthermore, previous studies on AWD irrigation indicate the potential for reducing greenhouse gas emissions from rice fields. However, such effects were not directly evaluated in this study and require further investigation to assess how automated precision irrigation may contribute to environmental sustainability in rice production systems.

## Figures and Tables

**Table 1:** Communication Performance Metrics of the Automated Irrigation System.

Parameter	Value
Total GPRS transmissions	10.29 million
Successful GPRS uploads	9.86 million
Observed GPRS failures	0.43 million
Temporary transmission failures	4.16%
Average GPRS latency	17–30 s
Average GSM latency	11 s
SMS commands sent	210
SMS commands received	210
Missed commands	Nill
Each Downtime events	1–2 min
Downtime events	20–30 times per day
Average Cumulative downtime	55 min per day
Total duration of analysis	100 days

**Table 2:** Yield components as affected by different irrigation treatments.

Treatment	Tiller/m^2^	Panicle/m^2^	Grain/Panicle	Thousand Grain Weight, gm
CF	265.7 b	247.7 b	132.0 a	23.7 a
IRRISAT	269.0 b	254.3 ab	133.0 a	23.6 a
IRRI35	281.0 a	261.0 a	130.7 a	23.7 a
IRRI25	249.7 c	232.0 c	130.7 a	23.8 a
LS	**	**	NS	NS
CV	1.36	1.86	-	-
LSD_0.05_	10.2	13.1	-	-

Common letters within the column do not differ statistically at either the 1% or 5% level of probability (analyzed using R); ** = Statistically significant at 1% level of probability, LS = Level of significance, NS = Not significant.

**Table 3:** Water use efficiency for different irrigation treatments.

Treatments	Land Preparation, mm	Effective Rainfall, mm	Irrigation, mm	Total Water Received, mm	Water Use Efficiency, kg ha^−1^ mm^−1^
CF	150	125	867 a	1142 a	6.7 c
IRRISAT	150	125	640 b	915 b	8.6 b
IRRI35	150	125	547 c	822 c	9.4 a
IRRI25	150	125	457 d	732 d	9.7 a
LS	-	-	**	**	**
CV	-	-	1.27	0.88	2.34
LSD_0.05_	-	-	22.48	83.9	0.57

Common letters within the column do not differ statistically at either the 1% or 5% level of probability (analyzed using R); ** = Statistically significant at 1% level of probability, LS = Level of significance.

**Table 4:** Energy saved by different irrigation treatments.

Treatments	Energy Consumption, kWh ha^−1^	Energy Saved Over CF, %
CF	932.5 a	-
IRRISAT	695.8 b	25.4
IRRI35	588.0 c	36.9
IRRI25	497.5 d	46.6
LS	**	-
CV	1.62	-
LSD_0.05_	31.05	-

Common letters within the column do not differ statistically at either the 1% or 5% level of probability (analyzed using R), ** = Statistically significant at 1% level of probability, LS = Level of significance.

**Table 5:** Perched water depth and drying days of different irrigation treatments.

Treatments	Perched Water Depth	Each Drying Period	Number of Irrigation Events	Time to Saturate the 0–15 cm Soil
CF	+5 cm	-	16	-
IRRISAT	−3–5 cm	1 to 2 days	14	1.7 h
IRRI35	−19–22 cm	4 to 5 days	11	4 h
IRRI25	−38–41 cm	7 to 8 days	10	6.7 h

**Table 6:** Component-wise cost of the automated precision irrigation treatment.

System	Component	Cost (US$)
IoT and sensor control panel for each plot	Arduino UNO Micro controller	9.0
	Mini GSM/GPRS module	9.0
	DC-DC Buck Converter 5 V 5 A	3.0
	Small Solar Panel 10 w	5.0
	Solar charge controller	5.0.
	12 v Battery 9 AH	12.0
	Ultrasonic sensor	2.0
	Soil moisture sensor 3 Pcs	15.0
	Real-time clock module	2.0.
	Electric Box for enclosure	5.0
	Connecting wires and fittings	1.0
Automatic Irrigation Control Gate for each plot	Automatic sliding gate wooden frame	25.0
	Linear Actuator 12-inch 12 v	45.0
	12 V two-way motor driver module	4.0
Subtotal		142.0
Automatic Pump Control Unit	Arduino UNO Micro controller	9.0
	Mini GSM/GPRS module	9.0
	DC-DC Buck Converter 5 V 5 A	3.0
	1 Channel relay module for Pump	3.0
	Electric Box	5.0
	Connecting wires and fittings	1.0
Communication cost	GSM and internet package/season	10.0
Maintenance	Wiring and servicing	2.0
Total		184

## Data Availability

The datasets presented in this article are not readily available because the data will be made available strictly for academic purposes upon justification. Requests to access the datasets should be directed to Y.D. (corresponding author).

## Abbreviations

The following abbreviations are used in this manuscript:

AWD	Alternate wetting and drying
IoT	Internet of Things
BRRI	Bangladesh Rice Research Institute
VWC	Volumetric Soil Water Content
CF	Continuous Flooding
